# Immune response effects of diverse vaccine antigen attachment ways based on the self-made nanoemulsion adjuvant in systemic MRSA infection†

**DOI:** 10.1039/c8ra00154e

**Published:** 2018-03-14

**Authors:** Liu-yang Yang, Chao Wei, Yun Yang, Ya-nan Tong, Sha Yang, Liu-sheng Peng, Qian-fei Zuo, Yuan Zhuang, Ping Cheng, Hao Zeng, Quan-ming Zou, Hong-wu Sun

**Affiliations:** National Engineering Research Center of Immunological Products, Department of Microbiology and Biochemical Pharmacy, College of Pharmacy, Third Military Medical University of Chinese PLA 30 Sha Ping Ba Gaotanyan Street Chongqing 400038 P. R. China sunhongwu2001@163.com +86-023-68752377 +86-023-68752377

## Abstract

Nanoemulsion adjuvants-based vaccines have potent induced immune responses against methicillin-resistant *Staphylococcus aureus* (MRSA) infection. However, the efficacies and immune responses of different antigen-attaching ways on self-made nanoemulsion adjuvants remain unknown. In this study, we designed three formulations of nanoemulsion adjuvants (encapsulation, mixture, and combination) to explore their immune response-enhancing effects and their underlying mechanism in a systemic infection model of MRSA. Our results showed that the three nanoemulsion-attachment ways formulated with a fusion antigen of MRSA (Hla_H35L_IsdB_348–465_) all improved humoral and cellular immune responses. When compared with the mixture and combination formulations, the nanoemulsion-encapsulation group effectively promoted the antigen uptake of dendritic cells (DCs) *in vitro*, the activation of DC in draining lymph nodes and the delayed release of antigen at injection sites *in vivo*. Moreover, the encapsulation group induced a more ideal protective efficacy in a MRSA sepsis model by inducing more potent antibody responses and a Th1/Th17 biased CD4^+^ T cell response when compared with the other two attachment ways. Our findings suggested that the encapsulated formulation of vaccine with nanoemulsion adjuvant is an effective attachment way to provide protective immunity against MRSA infection.

## Abstract 2

Encapsulated formulation of nanoemulsion vaccine induced more potent immune responses against methicillin-resistant *Staphylococcus aureus* (MRSA) infection, compared with combination and mixture attachment ways.

## Graphical abstract

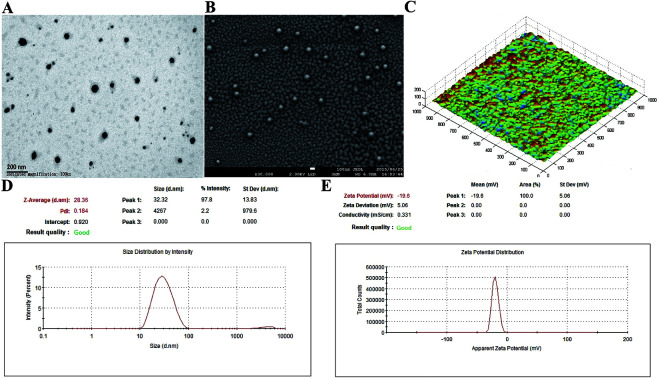

## 1. Introduction

With increasing number of Methicillin-resistant *Staphylococcus aureus* (MRSA) strains emerging in hospitals, there is an urgent need to develop an effective vaccine to combat MRSA infection.^1^ As an ingredient of vaccines, adjuvants are indispensable for enhancing and directly inducing robust and extensive adaptive immune responses associated with vaccine antigens.^2,3^ Currently, many public and private initiatives have designed *S. aureus* vaccines containing adjuvants (such as aluminium salts and emulsion adjuvants) that are in pre-clinical or clinical trials.^4^ Aluminum salts are widely used as vaccine adjuvants due to their potential to increase antigen immunogenicity.^5–8^ Emulsion adjuvants (such as AS03 by GlaxoSmithKline and MF59 by Novartis) have been approved in the USA and the European Union.^9^ Recently, NB-001, a novel emulsion adjuvant, has been registered in the USA and is undergoing Phase III clinical trial.^10^ However, certain disadvantages accompany the use of these adjuvants: (1) the aluminium adjuvant results in weak cellular immune responses^8,11–13^ and (2) the emulsion adjuvant shows thermodynamic instability because of its large size (>160 nm).^14^ Therefore, it is essential to identify a novel adjuvant, which can greatly increase the immune responses.

Nanotechnology is widely used in vaccine development for its ability to improve the immunogenicity of antigens.^15^ Several nanovaccine delivery systems (such as nanoemulsions and nanoparticles) have shown significant potential for improving immune responses.^16^ In particular, nanoemulsions-based vaccines have displayed robust protective efficacy in bacterial vaccine development.^13,14^ Our recent study has also indicated that a nanoemulsion-based adjuvant potently induces strong immune responses and can effectively improve the stability of bovine serum albumin or recombinant protein Hla_H35L_IsdB_348–465_ (derived from the Hla and IsdB genes).^17,18^

The efficacy of a vaccine is affected by several factors, such as the physicochemical properties of the micro-particles or nanoparticles (*e.g.*, particle size, surface charge, and hydrophobicity), administration routes and release kinetics.^19^ Antigen release kinetics are particularly determined by the different approaches used for attaching the antigens, such as conjugation, encapsulation, absorption, combination and mixture.^20^ Antigens encapsulated into nanoparticles may lead to slow antigen release, which then enhance the immune responses.^21^ Studies have shown that antigen-encapsulated nanoparticles are more effective in increasing the immune responses because the antigen is deposited at the injection site.^22^ However, some other studies show that antigen entrapment within nanoparticles may lead to a weak immune response because the antigen may not be released at the right time, concentration or location.^23,24^ A physical mixture of the antigen with nanoparticles is generally based on charge or hydrophobic interactions. However, the effects of the different antigen-attaching ways used for nanoemulsion adjuvant on the immune responses remain unknown.

Similar to the nanoparticles, antigens formulated with a nanoemulsion adjuvant include three attachment ways (encapsulation, mixture and combination). Various antigen attachment ways may affect the kinetics of antigen exposure to the immune system and may lead to different antigen-specific immune responses.^25^ However, relatively limited attention has been focused on the antigen attachment ways in nanoemulsion adjuvants. In order to explore the immune responses of the three antigen attachment ways, we identified the strength and type of immune responses induced by the different approaches based on our self-made nanoemulsion adjuvant and recombination protein Hla_H35L_IsdB_348–465_.

## 2. Materials and methods

### 2.1 Mice and recombination protein

Balb/c mice (SPF grade, 6–8 weeks, female) and nude mice (SPF grade, 6–8 weeks, female) were both obtained from Beijing HFK Bioscience Co., Ltd. (Beijing, China). All the animal experiments were performed in accordance with the Guide for the Care and Use of Laboratory Animals after being approved by the Animal Ethical and Experimental Committee of the Third Military Medical University of PLA, Chongqing, China. All mice that underwent surgery were treated with sodium pentobarbital anaesthesia to minimize suffering.

The MRSA recombination protein Hla_H35L_IsdB_348–465_ (1 mg mL^−1^), derived from IsdB and Hla with a 13.843 min peak time and 99.4% purity (HPLC), was prepared according to our previous methods.^26^ The endotoxin level of Hla_H35L_IsdB_348–465_ was at acceptable levels (<2.5 pg μg^−1^).

### 2.2 Preparation and characterization of the nanoemulsion adjuvant vaccine

The three attachment ways (encapsulation, mixture, and combination) for the nanoemulsion adjuvant vaccine were prepared according to our previously reported low energy emulsification methods^18,26^ and are shown in Fig. S1.† Briefly, surfactant EL 35® (Cremophor-35, polyethoxylated castor oil, BASF, Mumbai, India) and co-surfactant propylene glycol (Shanghai Chemical Regent, Shanghai, China) were mixed at a ratio of 4 : 1 (w/w). Oil phase-isopropyl myristate (IPM, Croda, Goole, UK) was added to the mixture to obtain a 9 : 1 (Smix–IPM) ratio at a concentration of 200 μg mL^−1^ protein in the encapsulation nanoemulsion vaccine. Next, the mixture was added dropwise under gentle agitation until the nanoemulsion was transparent and flowed easily. A blank nanoemulsion (BNE) was prepared according to the same methods without the addition of the antigen protein. The mixture formulation of the nanoemulsion vaccine was stirred for 2 h at 16 °C after adding the same concentration of antigen to the BNE. The combination formulation was stirred for 2 h at 16 °C after adding the encapsulation and the mixture formulations at a 1 : 1 volume ratio.

The morphology of the samples was subsequently characterized by scanning electron microscopy (SEM; JEM-6700F, JEOL Ltd., Tokyo, Japan) and transmission electron microscope (TEM; JEM-1230, JEOL Ltd., Tokyo, Japan). The molecular structure was observed on an AFM IPC-208B (Chongqing University, China). The average size and zeta potential were measured on a Nano-ZS 90 (Malvern Instruments Ltd., Malvern, UK).

### 2.3 Immunisation procedure and MRSA challenge infection

The immunisation procedure was consistent with a previous study.^18^ As shown in Fig. S2B,† balb/c mice were immunised with 30 μg of protein *via* intramuscular injection on days 0, 7 and 14. Histidine was used as the control.

For the sepsis infection model, balb/c mice were intravenously infected with 1 × 10^9^ CFUs MRSA 252 (lethal infection dose) or 2.5 × 10^8^ CFUs MRSA 252 (infection dose) on day 28 or on day 21, respectively. For the survival rate, the mice were monitored for 14 days after infection. For bacterial burdens, the organs (blood, spleens, and kidneys) were harvested on days 1 and 3 post-infection (Fig. S2A†).

For histopathology, whole kidneys were fixed with 10% phosphate-buffered formalin and embedded in paraffin. Four-micrometer-thick sections were prepared and stained with hematoxylin and eosin (HE) for microscopic examination. Each section was given a score of 0–4 (0: no abnormality; 1: area of renal tubular interstitial lesion <5%; 2: 5–25%; 3: 25–75%; and 4: >75%).

### 2.4 Enzyme linked immunosorbent assay (ELISA)

Sera were collected from all mice on days 7, 14 and 24 and stored at −80 °C for further analysis. Serum samples were used as primary antibodies to coat the wells of microtitre plates overnight at 4 °C. Secondary antibodies included HRP-conjugated goat anti-mouse IgG, anti-mouse IgG1 and anti-mouse IgG2a (Santa Cruz, CA, USA). The antibody responses were monitored at OD450.

### 2.5 Splenocyte proliferation assay and cytokine assay

Splenocyte proliferation assay was performed using CCK-8 kits (Dojindo, Japan). Splenocytes were suspended in complete media (RPMI 1640 with 10% FBS) at a concentration of 2.5 × 10^6^ cells per mL. The cells were stimulated with or without 10 μg mL^−1^ of protein at 37 °C for 3 days. The results were expressed as the proliferation index (PI), which was calculated based on the following formula: PI = OD (450 nm) for stimulated cultures/OD (450 nm) for non-stimulated cultures.

The supernatants were collected for cytokine assay and the levels of IFN-γ, IL-4 and IL-17A were determined through ELISA using mouse IFN-γ ELISA, IL-4 ELISA and IL-17A ELISA kits (Biolegend, San Diego, CA, USA), respectively.

### 2.6 Determination of memory T cell responses by flow cytometry

The percentage of memory T cells in the splenocytes was measured using a FACS Canto II flow cytometer (BD biosciences, USA). Splenocytes (1 × 10^7^ cells per mL) were stimulated with protein (10 μg mL^−1^) and incubated for 3 days. Then, the splenocytes were measured after labelling with fluorochrome-conjugated anti-mouse PE-cy7-CD4, PE-CD44, or APC-CD62L antibodies (Sungene, Tianjin, China). All data were analysed using FlowJo software (version 7.6; Oregon, USA).

### 2.7 Antigen uptake by BMDCs

The BMDC were prepared from bone marrow based on a method described previously.^27^ Briefly, bone marrow cells were primarily isolated from the femurs and tibias of female mice. The obtained cells were seeded in 6-well culture plates at 10^7^ cells per well in 1640 medium supplemented with 10% foetal bovine serum, 2 mM l-glutamine and 50 μM 2-mercaptoethanol. To induce DC differentiation, 10 ng mL^−1^ of granulocyte macrophage colony-stimulating factor (GM-CSF) (Preproch, USA) and 10 ng mL^−1^ of IL-4 (Preproch, USA) were added. All cultures were fed by replacing half of the medium and cytokines on days 3 and 5. On day 7, the cells were collected for further use.

The green fluorescence protein (GFP) uptake by the BMDCs were performed by a method previously described.^28^ First, the BMDCs were seeded overnight at 37 °C in 6-well plates at a density of 1 × 10^6^ cells per well. The media was replaced with fresh media containing 20 μg of the GFP proteins (encapsulation, combination and mixture). The cells were incubated at 37 °C for 30 min, washed with PBS, fixed in 4% paraformaldehyde, and dried with 1,1′-dioctadecyl-3,3,3′,3′-tetramethylindocarbocyanine perchlorate (Dil, 10 μg mL^−1^, Beyotime Institute of Biotechnology, Wuhan, China). The cells were imaged on a TCS-SP5 laser scanning confocal fluorescence microscope (Leica, Wetzlar, Germany) (green fluorescence, ex = 549 nm and em = 565 nm).

### 2.8 Expression of MHC and co-stimulatory molecules on DC in draining lymph nodes

As shown in Fig. S2C,† popliteal lymph nodes were harvested and processed into single cell suspension. The cells were stained with a mixture of anti-mouse antibodies (Percp-cy5.5 anti-CD11c, APC anti-MHC I, FITC anti-MHC II, and PE anti-CD86; eBioscience, USA). Expression of MHC I, MHC II, and CD86 on the CD11c^+^ DCs was determined using a FACS Canto II flow cytometer (BD biosciences, USA).

### 2.9 Frequency of follicular helper CD4^+^ (Tfh) cells in draining lymph nodes

The cells were stained with a mixture of anti-mouse antibodies (Percp-cy5.5 anti-CD4, APC anti-CXCR5, and PE anti-PD-1; eBioscience, USA). The percentage of Tfh cells (CD4^+^CXCR5^hi^PD-1^hi^) was determined using a FACS Canto II flow cytometer (BD biosciences, USA).

### 2.10 Antigen persistence at injection sites

Fifteen nude mice were divided into five groups (GFP group, three different GFP attachment types, and MF59, *n* = 3) and injected subcutaneously (left hind leg) or intramuscularly (right hind leg) with 100 μL of the different formulations of the nanoemulsion adjuvant vaccines containing 1 mg mL^−1^ GFP protein. Antigen persistence at the injection sites was documented using a Carestream FX PRO *in vivo* imaging system (IVIS Spectrum, Perkin Elmer, Massachusetts, USA) at the indicated time points (ex = 730 nm and em = 790 nm). Carestream molecular imaging software was used to quantify the sum of the fluorescence intensity at the injection sites.

### 2.11 Statistical analysis

Data were presented as the mean ± standard deviation (SD). The survival rates were analysed using Kaplan–Meier survival curves. Antibody responses were analysed using the Student's *t*-test. The bacterial burdens and severity scores were analysed using the non-parametric Mann–Whitney test. All data were analysed using GraphPad Prism 5.0 software (San Diego, CA, USA). The significant differences were expressed as *P < 0.05, **P < 0.01, and ***P < 0.001.

## 3. Results

### 3.1 Design and characterization of the nanoemulsion adjuvant vaccine

**Fig. 1:**
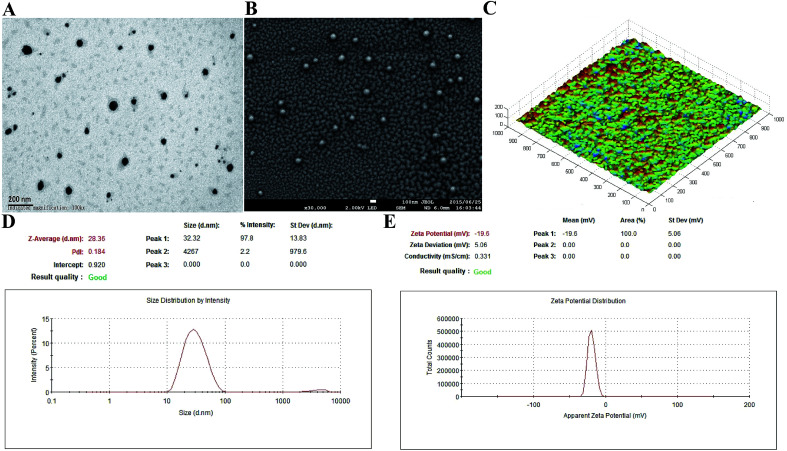
Characterization of the encapsulation attachment way. Transmission electron micrographs (A), scanning electron micrographs (B) and atomic force micrographs (C) of the encapsulation attachment way. The size distribution (D) and zeta potential (E) were detected using nano ZS.

In the present study, 200 μg mL^−1^ of antigen protein (Hla_H35L_IsdB_348–465_) in the nanoemulsion adjuvant vaccine was prepared through three different ways (encapsulation, mixture and combination; Fig. S1†). The encapsulation attachment way yielded a high encapsulation efficiency (91.3%) and drug loading (450 μg mL^−1^). The particles prepared *via* the encapsulation attachment way appeared dark under TEM (Fig. 1A). The SEM images also show that the encapsulated formulation yielded spherical particles with uniform distribution and the particle surfaces were rough with sags and crests (Fig. 1B). AFM images indicated that most of the particles exhibited approximately spherical morphology with mean diameters of nearly 30 nm (Fig. 1C). No morphology differences were observed in the three vaccine antigen attachment ways (data not shown). The average particle size of the droplets prepared by the encapsulation way was 28.36 nm (Fig. 1D). The average particle sizes of the droplets prepared by the mixture and combination way were 29.26 nm and 30.34 nm, respectively (Fig. S3A†). The PdI values were all less than 0.3 (0.184, 0.270 and 0.210), suggesting that all prepared three nanoemulsion formulations exhibit good narrow size distributions.^29^ The average zeta potential of the encapsulation, mixture and combination way were −19.60 mV, −21.60 mV and −21.56 mV, respectively, all ranging from −30 to 30 mV (Fig. 1E and S3B†). Thus, the three vaccine antigen attachment ways all showed good size distributions and stable zeta potentials.

### 3.2 Antibodies production by the three vaccine antigen attachment ways

**Fig. 2:**
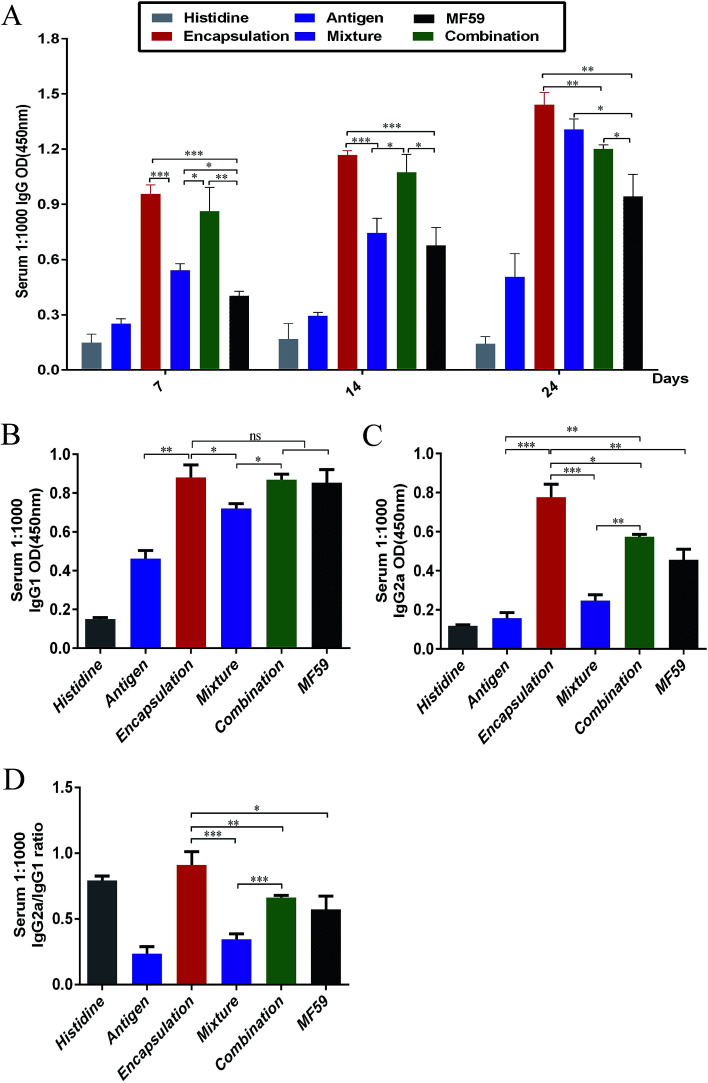
Antibody responses by the three vaccine antigen attachment ways. (A) Serum (*n* = 6) were taken at days 7, 14 and 24. IgG responses were detected through ELISA. (B and C) Serum (*n* = 6) were taken at days 24. The responses of IgG1 and IgG2a were detected through ELISA. Antibody response is expressed as the mean absorbance at 450 nm ± SD. (D) IgG2a/IgG1 ratio was calculated. *P < 0.05; **P < 0.01; ***P < 0.001.

To analyse the antibody responses induced by the three formulations, the IgG and IgG sub-classes in serum were detected using ELISA as shown in Fig. S2A.† Among the six groups, the encapsulation group induced the most potent IgG responses on days 7, 14 and 24 (Fig. 2A). The IgG levels of the encapsulation and combination group were significantly higher than the MF59 group on days 7 (*P* < 0.001; *P* < 0.001), 14 (*P* < 0.001; *P* < 0.05) and 24 (*P* < 0.001; *P* < 0.05). In addition, the mixture group induced more powerful IgG responses than the MF59 group on day 24 (*P* < 0.05). Furthermore, the encapsulation group induced significantly higher levels of IgG than the combination group (*P* < 0.05) and mixture group (*P* < 0.05) on day 24. Based on these results, all the three vaccine antigen attachment ways induced strong IgG responses and the encapsulation group induced the most potent IgG response among the three vaccine antigen attachment ways.

**Fig. 3:**
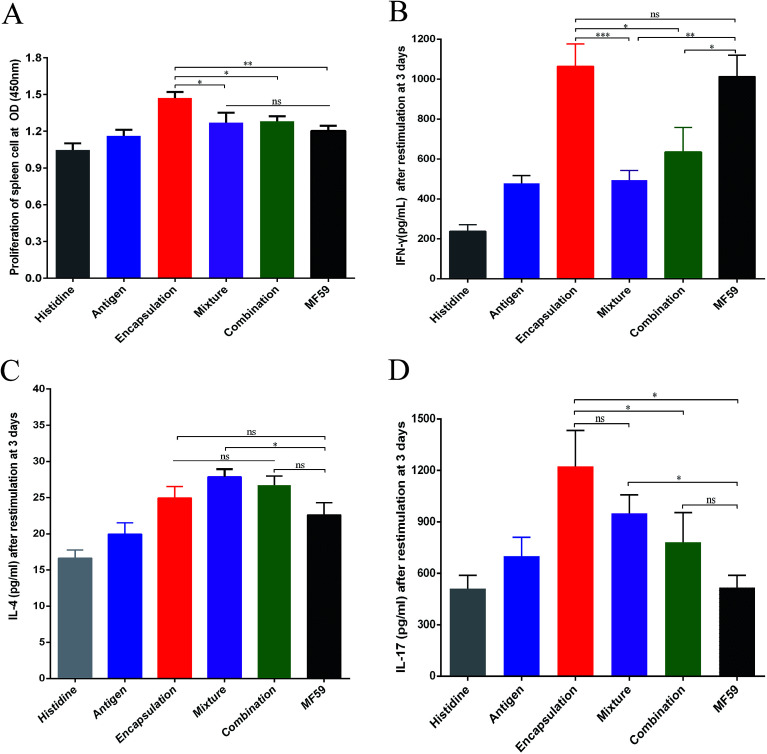
Cellular immune responses by the three vaccine antigen attachment ways. Splenocytes of immunised mice (*n* = 6) were stimulated with the antigen for 3 days. (A) Splenocyte proliferation assay was performed using a CCK-8 kit. (B–D) IFN-γ, IL-4 and IL-17 levels were detected through ELISA. The results are reported as the mean ± SD. *P < 0.05; **P < 0.01; ***P < 0.001.

Then, we detected the IgG1 and IgG2a responses in serum. The encapsulated formulation resulted in a more significant increase in the IgG1 levels when compared with the mixture group (*P* < 0.05) and it showed comparable levels with the combination group and MF59 group (Fig. 3B; *P* > 0.05). In addition, the IgG2a response induced by the encapsulated formulation was the most potent among all groups (Fig. 3C) and it was also significantly higher than those of the mixture, combination and MF59 groups (*P* < 0.001; *P* < 0.05; *P* < 0.01). However, the mixture group induced the lowest levels of IgG2a among the three vaccine antigen attachment ways. As shown in Fig. 3D, the IgG2a/IgG1 ratio in the encapsulation group was significantly higher than the mixture, combination or MF59 groups (*P* < 0.001; *P* < 0.01; *P* < 0.05). Collectively, the encapsulated formulation induced strong IgG responses and a significant increase in the IgG2a subgroups.

### 3.3 Cellular immune responses induced by the three vaccine antigen attachment ways

**Fig. 4:**
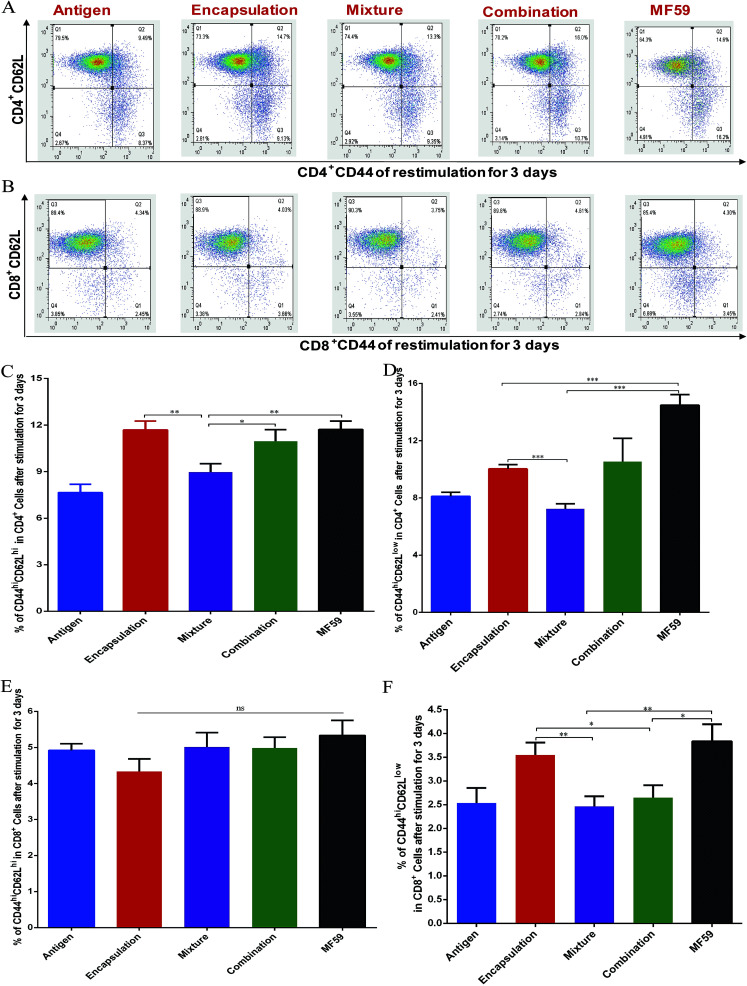
Frequency of memory T cells induced by the three vaccine antigen attachment ways. Balb/c mice (*n* = 6) were immunised as previously described. On day 24, the splenocytes (1 × 10^7^ cells per mL) were stimulated with antigen (10 μg mL^−1^) for 3 days. FACS plots in (A and B) are representative of the mean percentages of 6 mice in each group. The frequency of CD44^hi^CD62^hi^ CD4^+^ T cells (C), CD44^hi^CD62^low^ CD4^+^ T cells (D), CD44^hi^CD62^hi^ CD8^+^ T cells (E) and CD44^hi^CD62^low^ CD8^+^ T cells (F) were measured using flow cytometry. Data in (C), (D), (E), and (F) are expressed as the mean ± SD. *P < 0.05; **P < 0.01; ***P < 0.001.

To evaluate the cellular immune responses of the various nanoemulsion formulations, we performed splenocyte stimulation tests. As shown in the Fig. 4A, the encapsulation group achieved the highest splenocyte lympho-proliferation and this proliferation was significantly higher than that of the mixture, combination and MF59 group (*P* < 0.05, *P* < 0.05, *P* < 0.01). However, there were no differences in the splenocyte lympho-proliferation between the mixture, combination and MF59 groups (*P* < 0.05).

To evaluate the effects of the various nanoemulsion formulations on the systemic cytokine levels more concretely, we then measured the levels of IFN-γ, IL-4 and IL-17 produced by the splenocytes after stimulation using the recombinant proteins. We observed that the IFN-γ level of the encapsulation group was higher than those of the mixture and combination group (Fig. 4B; *P* < 0.001; *P* < 0.05). In addition, the three formulations induced comparable amounts of IL-4 and the mixture group induced the highest level of IL-4 without any statistical difference (Fig. 4C). It seemed that the mixture group induced a Th2 biased immune response (Fig. 2D and 3C). Furthermore, after stimulation, the splenocytes in the encapsulation group produced greater amounts of IL-17A than the combination and MF59 group (Fig. 4D; *P* < 0.05). These data confirmed that the encapsulation attachment way induced a powerful cellular immune response to skew a Th1/Th17 response.

### 3.4 Central memory and effector memory levels induced by the three vaccine antigen attachment ways

**Fig. 5:**
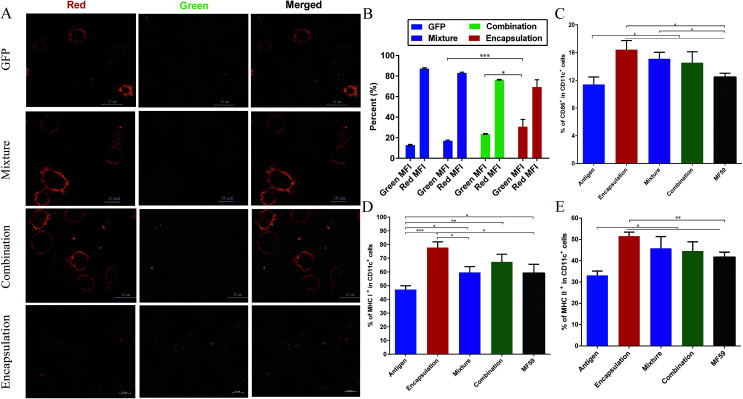
Antigen uptake by DCs and DC activation in the draining lymph nodes. (A) CLSM images of BMDCs after 30 minutes of incubation with the naïve GFP protein, GFP-combination nanoemulsion, GFP-mixture nanoemulsion and GFP-encapsulation nanoemulsion. For each panel, the images from left to right show Dil stained cell members by Lyso-Tracker (red), GFP fluorescence (green) and the overlays of the two images (red and green). (B) Results of the fluorescence intensity percentage (*n* = 3) are expressed as the mean ± SD. Balb/c mice (*n* = 6) were intramuscularly vaccinated with the different vaccine formulations. At 8 days after immunisation, the mice were euthanized and the popliteal lymph nodes were isolated. The percentage of CD86 (C), MHC I (D) and MHC II (E) expression on the CD11c^+^ DCs were determined using flow cytometry. Data are expressed as the mean ± SD. *P < 0.05; **P < 0.01; ***P < 0.001.

Memory T cells play key roles in inducing protective vaccine effects during subsequent bacterial infections.^30^ The central memory cells (CD44^hi^CD62L^hi^) and effector memory cells (CD44^hi^CD62L^low^) of CD4^+^ and CD8^+^ T cells were separated and analysed from immunised mice (Fig. 4A and B). The frequency of central-memory CD4^+^ T cells was similar between the encapsulation and MF59 groups. However, the frequency in both of these groups was significantly higher than that in the mixture group (Fig. 4A and C; *P* < 0.05). For the proportion of effector memory CD4^+^ T cells, the MF59 group induced a higher proportion than the encapsulation (*P* < 0.001) and mixture groups (*P* < 0.001). In addition, the encapsulation group induced higher CD4^+^ T levels than the mixture group (*P* < 0.001). The frequency of central memory CD8^+^ T cells was similar in the three vaccine antigen attachment ways (Fig. 5E). In addition, the encapsulation group and MF59 group induced a similar frequency of effector memory CD8^+^ T cells, which were significantly higher than the mixture group and combination group (Fig. 5F; *P* < 0.05). Therefore, the encapsulation group and combination group induced higher levels of memory T cells than the mixture group, suggesting that the encapsulation and combination group may provide better protection against the re-infection of MRSA.

### 3.5 Cellular uptake of nanoemulsion vaccine into DCs

As shown in Fig. 5A, among these three attachment ways, the encapsulated formulation and the BMDCs were highly co-localized 30 min after incubation. We demonstrated that the GFP encapsulation attachment way was efficiently internalized by the BMDCs after incubation, indicating the efficient cellular uptake of the encapsulated formulation. Furthermore, the GFP intensity percentage of the encapsulation group was significantly higher than those of the combination and mixture groups (*P* < 0.001, *P* < 0.05; Fig. 5B). This finding suggests that the encapsulation attachment way enhanced the cellular uptake of antigens into the DCs when compared to the other two attachment ways.

### 3.6 DC activation and follicular helper CD4^+^ T cells differentiation in the draining lymph nodes

It is well known that antigens are transported to the draining lymph nodes and this process can be affected by the different vaccine antigen attachment ways.^20^ Therefore, we assessed the activation of the DCs in the draining lymph nodes. MHC molecules (MHC I molecules, MHC II molecules) and co-stimulatory expression of CD86 on DCs from the draining lymph nodes were measured using flow cytometry. As shown in the Fig. 5C–E, when compared with the antigen group, the three vaccine antigen attachment ways and MF59 group all induced significantly higher expression of CD86, MHC I and MHC II at 8 days after vaccination (*P* < 0.05). Among the three formulations, only the encapsulated formulation elicited significantly higher expression levels of CD86, MHC I and MHC II than the MF59 group (*P* < 0.05). In addition, the mixture, combination and MF59 group induced comparable expressions of MHC I and MHC II. Therefore, the three vaccine antigen attachment ways can induce the effective expression of MHC and co-stimulatory molecules on the DCs in the draining lymph nodes and the encapsulated formulation can more efficiently induce DC activation.

Follicular helper T (Tfh) cells play a key factor for producing an efficient humoral immune response.^31^ To demonstrate whether the encapsulated formulation may be beneficial for generating an antibody response by improving Tfh cell activation, we then measured the frequency of Tfh cells in the draining lymph nodes of immunised mice. As shown in Fig. S4,† the encapsulated formulation induced a higher frequency of Tfh cells than the mixture and combination group (*P* < 0.01, *P* < 0.05).

### 3.7 Delayed release effects of the three formulations at the injection sites

**Fig. 6:**
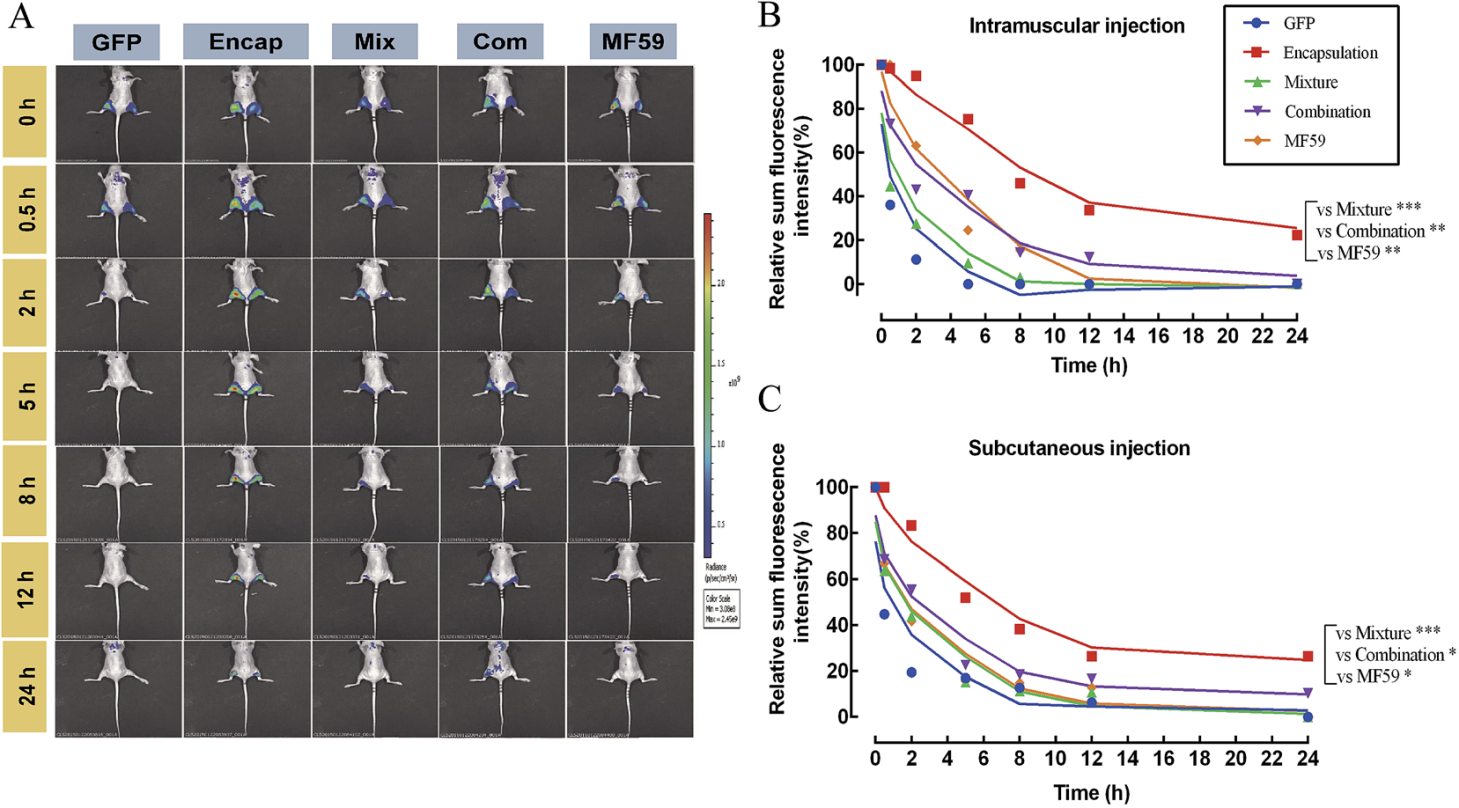
Antigen persistence and depot at the injection sites. Nude mice (*n* = 3) were injected subcutaneously (left hind leg) or intramuscularly (right hind leg) with 100 μL of the different formulations of the nanoemulsion adjuvant vaccines containing 1 mg mL^−1^ GFP protein. Antigen persistence at the injection sites was measured using a Carestream FX PRO *in vivo* imaging system. (A) Representative fluorescence images and (B and C) quantitative fluorescence intensity of the antigen persisting at the injection sites. Data are expressed as the mean ± SD (*n* = 3). *P < 0.05; **P < 0.01; ***P < 0.001.

We then tested the release effect of the three formulations at the injection sites. As shown in Fig. 6A, when compared with the other four groups, the encapsulated formulation showed a delayed release effect. The relative proportion of the sum of the florescence intensity for intramuscular and subcutaneous injections both confirmed that the encapsulated formulation significantly sustained the release effect of the antigen (Fig. 6B and C). Furthermore, we found that the encapsulation group showed a significant delayed release effect than the mixture, combination and MF59 group (*P* < 0.05). Therefore, the encapsulated formulation showed a significant delayed release effect and may greatly improve the immune response of the antigen.

### 3.8 Protective efficacy of various formulations in a MRSA sepsis model

**Fig. 7:**
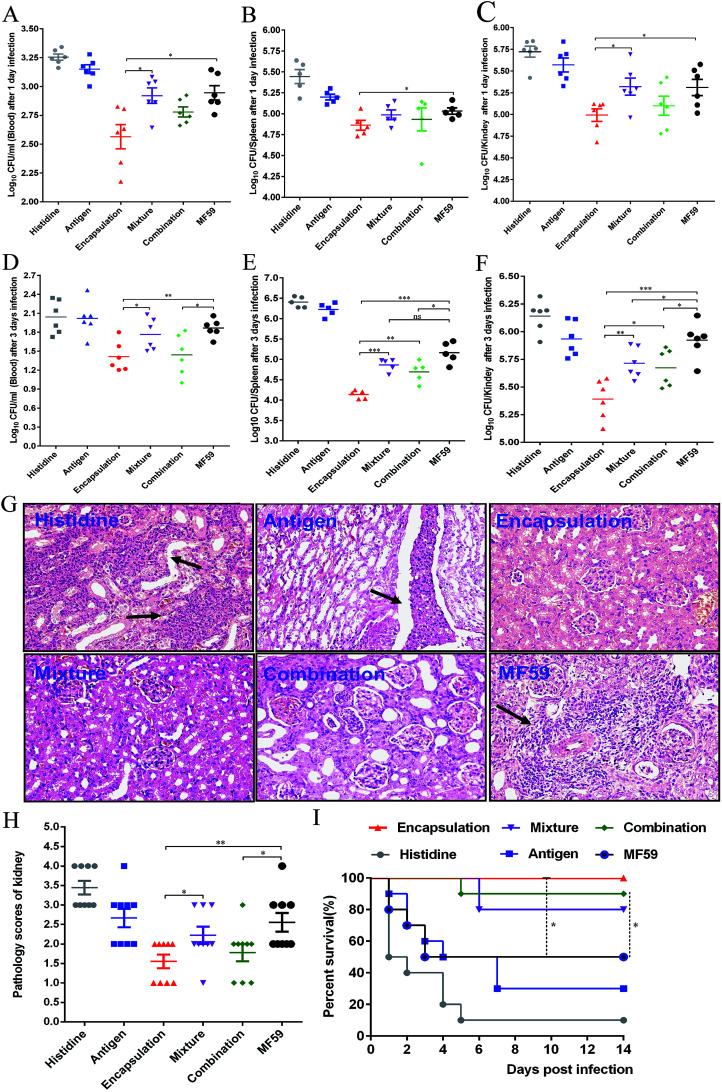
Protective effects of three vaccine antigen attachment ways in a MRSA sepsis model. Balb/c mice (*n* = 6) were immunised as previously described. At days 21, mice were infected with 2.5 × 10^8^ CFUs MRSA 252. At 1 and 3 days post-infection, the bacterial burdens in the blood (A, D), spleen (B, E) and kidneys (C, F) were measured. (G) At 3 days post-infection, the kidneys were collected and the representative histopathological sections are shown (magnification = ×400). Arrowheads indicate Staphylococcal abscesses. (H) Severity scores of the kidneys (*n* = 6) from the six immunised groups at 3 days post-infection are shown. Data are presented as scatter plots and the means ± SD are shown. (I) The mice (*n* = 10) were intravenously infected with MRSA 252 (1 × 10^9^ CFUs) and the survival rates were monitored for 14 days. *P < 0.05; **P < 0.01; ***P < 0.001.

To evaluate the protective efficacy, the bacterial burdens in the blood, spleen and kidneys were measured on days 1 and 3 post-infection. The three attachment ways and MF59 group all showed significant bacterial decrease in organs as compared to the histidine control group (Fig. 7A–F). In addition, the bacterial burdens of the encapsulation group were significantly lower than those of the mixture group (Fig. 7C–F; *P* < 0.05) and combination group (Fig. 7E and F; *P* < 0.05). Furthermore, when compared to the MF59 group, the encapsulation group significantly decreased the bacterial burdens in the blood, spleen and kidneys (Fig. 7A–F; *P* < 0.05). We also found that the bacterial burdens of the kidneys in the combination and mixture groups were lower than those of the MF59 group at 3 days post-infection (Fig. 7F). The results of the histological analyses and severity scores demonstrated that the kidneys from the mice in the encapsulation and combination group exhibited fewer renal abscesses and fewer inflammatory cell infiltrations than those in the MF59 group (Fig. 7G and H). In addition, these results also demonstrated that the encapsulation group showed less severity in the kidneys than the mixture group (Fig. 7H; *P* < 0.05). These data suggested that the encapsulation group showed a better protective effect than the mixture, combination and MF59 groups.

The survival rates were determined by administering a lethal dose of MRSA252. As shown in Fig. 7I, the survival rates of the three formulation groups (encapsulation: 100%; mixture: 80%; and combination: 90%) were higher than the MF59 group (50%) and antigen group (30%). Although the survival rates were comparable among the three formulations, the encapsulation group still induced the highest protective efficacy. Therefore, we concluded that immunisation with the encapsulated formulation induced a high protective effect in a MRSA infection model.

## 4. Discussion

Novel technologies or adjuvant formulate with antigens may pave the way for the development of vaccines.^4^ A nanoemulsion has been applied to improve antigen delivery and increase vaccine efficacy toward more selective and effective responses.^32^ Moreover, it enabled the application of 0.22 μm sterile filtration, making parenteral administration feasible due to terminal sterilization.^33^ Recent studies have shown that particles with smaller diameters ranging from 1 to 100 nm can be transported more rapidly and captured more efficiently by the DCs than larger particles (*i.e.*, greater than 160 nm) due to penetrating tissue barriers and quick transport to the draining lymph nodes.^34^ In our study, three formulations of a nanoemulsion adjuvant were designed with sizes ranging from 1 to 100 nm based on the recombinant protein Hla_H35L_IsdB_348–465_.

Several different antigen attachment ways of the adjuvant can be used to improve the immune response, including increasing the immunogenicity of weak antigens, enhancing the speed and duration of immune response and modulating antibody avidity, specificity, isotype, or subclass distribution.^21^ For the humoral responses, our results showed that the three attachment ways induced more potent antibody responses than the MF59 group. Moreover, the encapsulation group induced higher levels of IgG and IgG subclass than the mixture and combination group (Fig. 2A). Therefore, the encapsulation group induced the most potent humoral response among the three vaccine antigen attachment ways. Furthermore, the encapsulated formulation was significantly higher than the other two attachments in terms of accelerating the immune response towards a Th1 response according to the IgG2a/IgG1 ratio (*P* < 0.01). It is widely believed that the MF59 adjuvant is a Th1-dominant adjuvant and our results also suggested that the encapsulated formulation may also induce a strong Th1 immune response.

Adjuvants may affect the type of vaccine-induced cellular immune response.^35^ However, the type of immune response induced by the nanoemulsion attachment ways remains unknown. In our study, we found that the encapsulation nanoemulsion adjuvant induced higher IFN-γ and IL-17 levels than the combination and mixture ways (*P* < 0.05). The three attachment ways induced higher levels of IL-4 than the antigen group; however, the levels of IL-4 observed in the three groups were comparable (*P* > 0.05). Antigen-specific Th1/Th17 responses are consistent with the high survival rates in the different MRSA infection models and Th2 cell responses help to promote class switching and generate great amounts of functional antibody responses.^30,36^ Therefore, our results suggest that the encapsulated formulation induces strong cellular responses, which may play an important role in its protective efficacy.

Antigen delivery to antigen presenting cells (APC), such as macrophages and dendritic (DC), is a vital step for initiating an effective vaccine immune response.^37^ Many factors, such as the size, surface charge, shape, attachment ways, hydrophilic and receptor interactions can affect the antigen uptake by APC cells.^34^ In our study, we detected the antigen uptake by BMDC *in vitro*, the activation of DC in the lymph nodes and the antigen release at the injection site *in vivo*. First, we found that the DCs could uptake more particles in the encapsulation attachment way than those in the mixture and combination groups, implying that the encapsulated formulation may easily be captured by the APC cells. Second, the encapsulation attachment way significantly prolonged the antigen residence time at the injection site, which can efficiently induce the maturation and activation of the DCs and subsequent T cell activation. Third, the encapsulated formulation effectively induced DC activation in the draining lymph nodes, which consisted of higher frequencies of memory T cells and Tfh cells. The interaction between the MHC-peptide complexes (MHC-I and MHC-II) expressed on the APCs and TCR was required for T cell activation and antigen specificity.^38^ In addition, co-stimulatory molecules, such as CD86 and CD80, delivered by the same APC are required to trigger clonal expansion and differentiation of a naive T cell. In addition, Tfh cells are essential for helping B cells' activation and producing high avidity antibodies. In our study, the encapsulation attachment way induced higher DC activation (Fig. 5C–E) and a higher frequency of Tfh cells (Fig. S4†) in the draining lymph nodes. Collectively, these aforementioned results can explain why and how the encapsulated formulation elicited protective effects in the MRSA sepsis model with more potent antigen-specific immune responses than the other two formulations. Nevertheless, further investigations are required to understand the precise underlying mechanisms.

In summary, we designed three different attachment ways (encapsulation, mixture and combination) and systemically conducted comprehensive serological and cytological characterizations. As compared to the combination and mixture attachment ways, the encapsulated formulation induced higher protective efficacy in the MRSA sepsis model because it yielded more potent humoral immune responses and a Th1/Th17 biased cellular immune response. The enhanced immune response may be due to the delayed release effect of the antigen at the injection site, the long-term antigenic persistence and the effective induction of DC activation and Tfh cell differentiation in the draining lymph nodes. The identification of the encapsulated formulation vaccine should be a starting point to assist us in seeking more effective adjuvants in MRSA vaccines.

## Conflicts of interest

There are no conflicts of interest associated with the present work.

## Financial information

This study was supported by the National Major Scientific and Technological Special Project (No. 2016ZX09J16102-002), the National Natural Science Foundation of China (No. 31370932, No. 31670938, and No. 31600745), and the Natural Science Foundation Project Program of Chongqing CSTC (No. 2014jcyjA10107).

## Supplementary Material

RA-008-C8RA00154E-s001
