# Supplementary material for: Immune response effects of diverse vaccine antigen attachment ways based on the self-made nanoemulsion adjuvant in systemic MRSA infection
Source: RSC Adv. 2018 Mar 14;8(19):10425–36. doi: 10.1039/c8ra00154e (PMC9078882; doi:10.1039/c8ra00154e)
Supplement: RA-008-C8RA00154E-s001 [file RA-008-C8RA00154E-s001.pdf]

Electronic Supplementary Material (ESI) for RSC Advances.
This journal is © The Royal Society of Chemistry 2018

Supplementary Information

The immune response effect of diverse vaccine antigen attachment ways based on the self-made nanoemulsion adjuvant in the systemic MRSA infection

Liu-yang Yang, Chao Wei, Yun Yang, Ya-nan Tong, Sha Yang, Liu-sheng Peng, Qian-fei Zuo, Yuan Zhuang, Ping Cheng, Hao Zeng, Quan-ming Zou, Hong-wu Sun[#]

(National Engineering Research Center of Immunological Products & Department of Microbiology and Biochemical Pharmacy, College of Pharmacy, Third Military Medical University of Chinese PLA, Chongqing 400038, PR China)

*These authors contributed equally to this work.

#Correspondence: Hongwu Sun

National engineering research center for immunological products & Department of microbiology and biochemical pharmacy, College of pharmacy, Third military medical university of Chinese PLA, 30 Sha Ping Ba Gaotanyan Street, Chongqing, 400038, China.

Tel: +86-023-68752377;

Fax: +86-023-68752377.

Email: sunhongwu2001@163.com

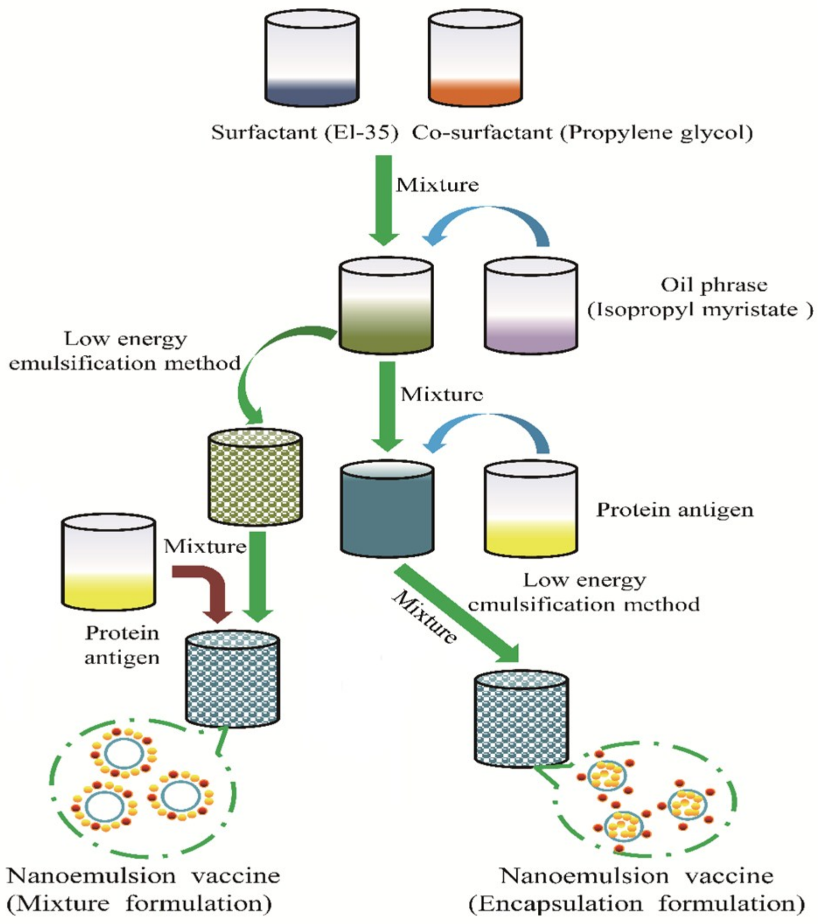

Figure S1. Schematic illustration of three different vaccine antigen attachment ways by the low energy emulsification methods.

The encapsulated formulation with 200μg/mL of the recombination antigen protein $Hla_{H35L}IsdB_{348-465}$ was prepared. The mixture attachment way was stirred for 2h at 16°C after adding the same concentration of $Hla_{H35L}IsdB_{348-465}$ and the BNE. The combination attachment way was stirred for 2h at 16 °C after adding half volume encapsulation and half volume mixture way of nanoemulsion vaccine.

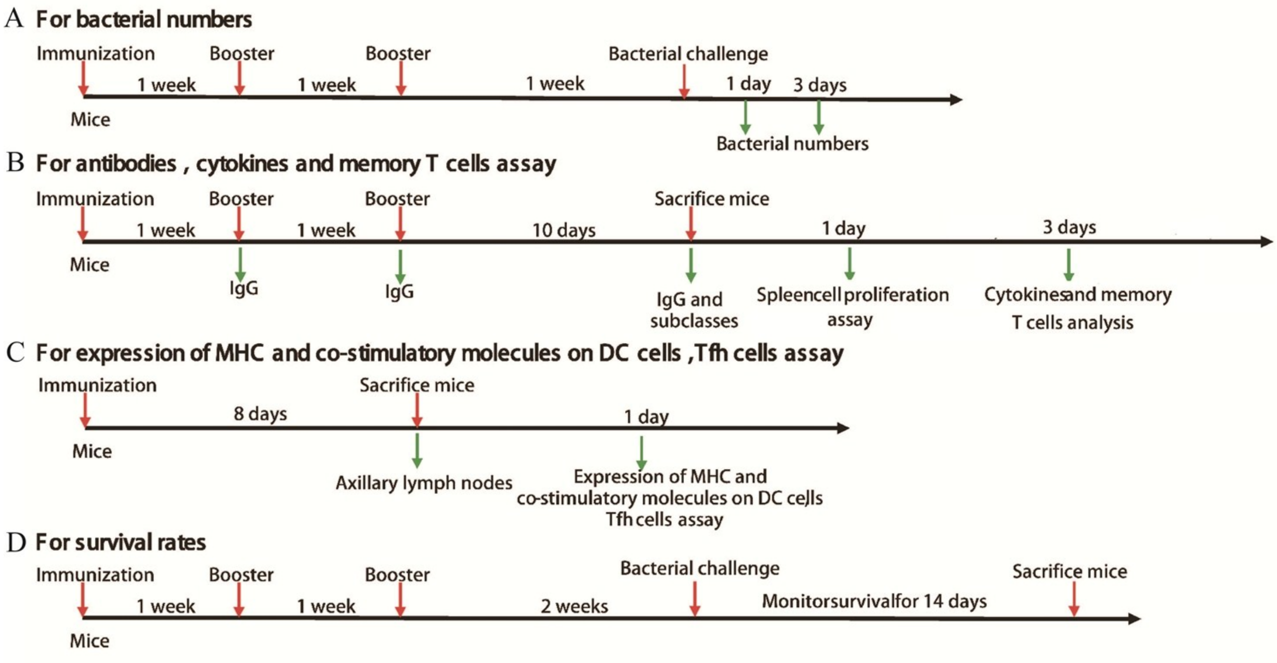

Figure S2. The animal experiment scheme.

Female blab/c mice (6–8 weeks old) were immunized by intramuscular injection into the upper quadriceps muscles three times on day 0, 7 and 14. (A) Bacterial burdens in organs were performed on 1 and 3 days post infection. (B) Antibody responses, spleen cell proliferation assay and memory T cell assay were performed on days 24, 25 and 27. (C) The expression of MHC and co-stimulatory molecules on DC cells and Tfh cells in draining lymph nodes were performed on 8 days after immunization. (D) Survival rates were performed on day 28 and monitored for 14 days.

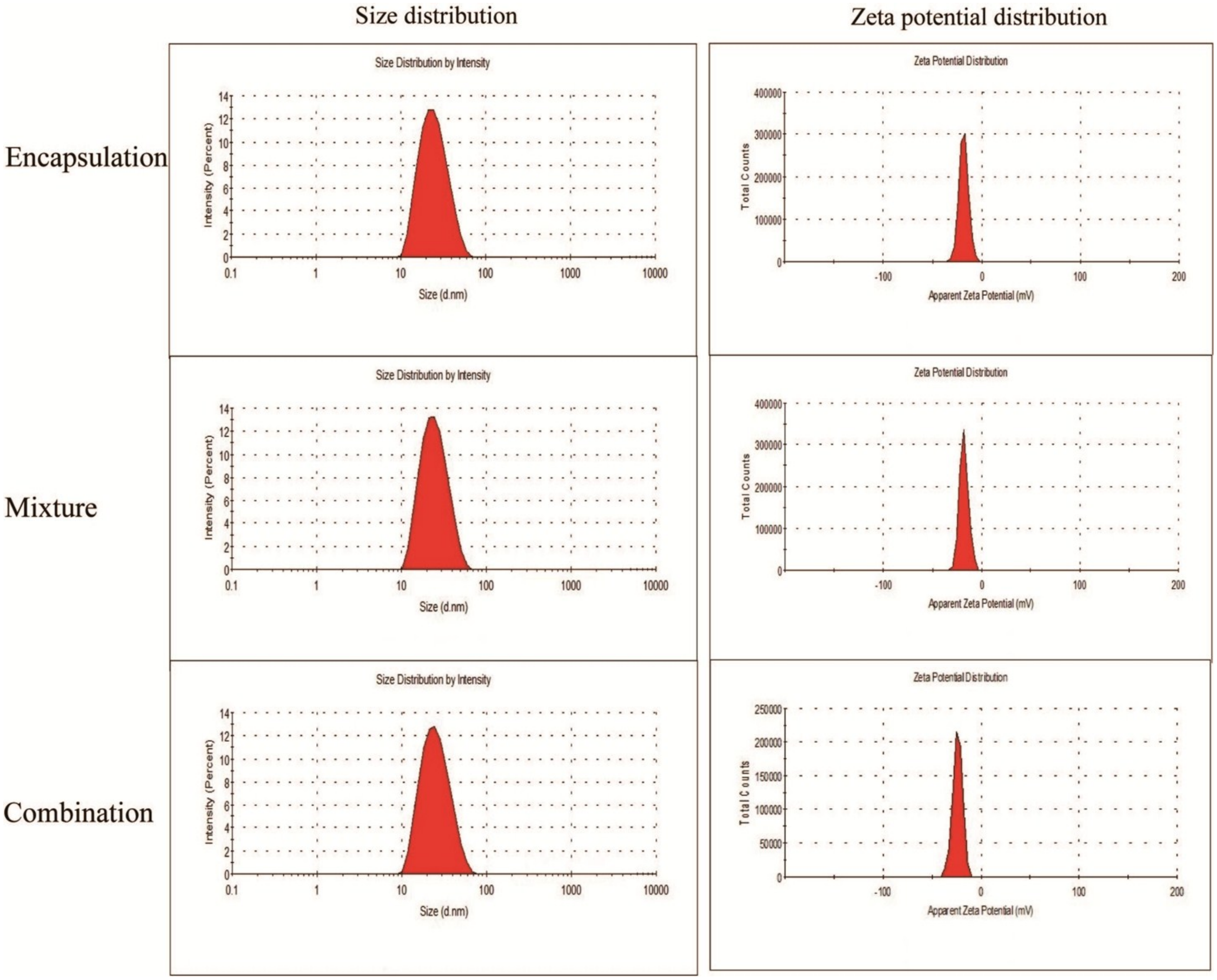

Figure S3. Size distribution and zeta potential distribution of three different nanoemulsion adjuvant vaccine (encapsulation, mixture and combination).

The size distribution and zeta potential distribution of encapsulation, mixture and combination attachment ways.

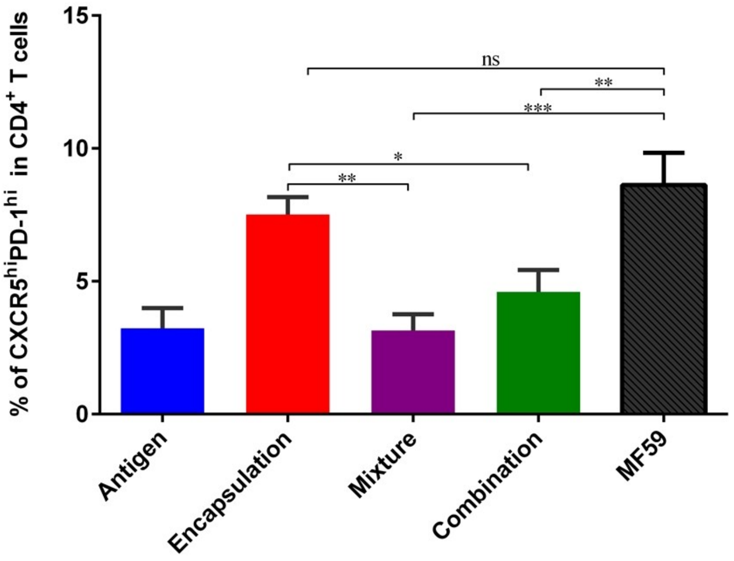

Figure S4. The frequency of follicular helper CD4^{+} T cells in the draining lymph nodes of immunized mice.

The popliteal lymph nodes of balb/c mice (n = 6) were isolated. The frequency of follicular helper CD4^{+} T cells (CD4^{+}CXCR5^{hi}PD-1^{hi}) was determined by flow cytometry. Data are expressed as the mean ± SD (n = 6).
